# Recent advances and challenges in potato improvement using CRISPR/Cas genome editing

**DOI:** 10.1007/s00425-022-04054-3

**Published:** 2022-12-23

**Authors:** Izabela Anna Chincinska, Magdalena Miklaszewska, Dorota Sołtys-Kalina

**Affiliations:** 1 Department of Plant Physiology and Biotechnology, University of Gdańsk, Wita Stwosza 59, 80-308 Gdańsk, Poland; 2 Department of Functional and Evolutionary Ecology, Division of Molecular Systems Biology (MOSYS), Faculty of Life Sciences, University of Vienna, Djerassiplatz 1, 1030 Vienna, Austria; 3 Plant Breeding and Acclimatization Institute-National Research Institute, Platanowa 19, 05-831 Młochów, Poland

**Keywords:** *Solanum tuberosum*, Polyploid plants, Tuber crops, Quality traits, Gene knockout, Transgene-free

## Abstract

**Main conclusion:**

Genome editing using CRISPR/Cas technology improves the quality of potato as a food crop and enables its use as both a model plant in fundamental research and as a potential biofactory for producing valuable compounds for industrial applications.

**Abstract:**

Potato (*Solanum tuberosum* L.) plays a significant role in ensuring global food and nutritional security. Tuber yield is negatively affected by biotic and abiotic stresses, and enzymatic browning and cold-induced sweetening significantly contribute to post-harvest quality losses. With the dual challenges of a growing population and a changing climate, potato enhancement is essential for its sustainable production. However, due to several characteristics of potato, including high levels of heterozygosity, tetrasomic inheritance, inbreeding depression, and self-incompatibility of diploid potato, conventional breeding practices are insufficient to achieve substantial trait improvement in tetraploid potato cultivars within a relatively short time. CRISPR/Cas-mediated genome editing has opened new possibilities to develop novel potato varieties with high commercialization potential. In this review, we summarize recent developments in optimizing CRISPR/Cas-based methods for potato genome editing, focusing on approaches addressing the challenging biology of this species. We also discuss the feasibility of obtaining transgene-free genome-edited potato varieties and explore different strategies to improve potato stress resistance, nutritional value, starch composition, and storage and processing characteristics. Altogether, this review provides insight into recent advances, possible bottlenecks, and future research directions in potato genome editing using CRISPR/Cas technology.

## Introduction

Potato is an important tuber-producing crop and a complex source of nutrients, carbohydrates, and dietary fiber. In 2020, potatoes were cultivated in 140 countries worldwide on an area of 16.5 million hectares, yielding 359 million tons (FAOSTAT 2022).

Potato originated from the Andean region of South America, namely Peru, Bolivia, and Northern Argentina. All tuber-bearing *Solanum* species are grouped into the section Petota, with four species of the cultivated potato: *S. tuberosum*, of which there are two cultivar groups, Andigenum (2, 3, and 4x) and Chilotanum (4x); *S. ajanhuiri* (2x); *S. juzepczukii* (4x); and *S. curtilobum* (5x). A total of 107 wild potato species have been described (Spooner et al. 2014).

Potato tubers are a staple food in many countries worldwide, and the challenges associated with climate change make it imperative to increase potato yield sustainably. It is estimated that global warming will impose a shift in both the locations of potato cultivation and the timing of potato planting, as well as the use of late-maturing cultivars (Hijmans 2003). Potato is susceptible to high temperatures, and this particularly affects tuberization initiation and leads to yield reduction. Thus, heat-tolerant cultivars are especially needed to alleviate the negative effects of global warming, especially in subtropical regions. However, adequate cultivation time and the proper selection of varieties may allow for yield increases in countries with a temperate climate, such as Finland and Great Britain (Hijmans 2003).

The responses of potato cultivars to abiotic stresses such as drought, salinity, low temperature/frost, and high temperature are variable, particularly with regard to the specific genotype and the duration and severity of the stress (Demirel et al. 2020). Biotic stresses such as pests and diseases generate yield losses of up to 88% (Kolychikhina et al. 2021). Potato plants suffer from diseases caused by several pathogens including the oomycete *Phytophthora infestans* (late blight), the potato leafroll virus (PLRV), the potato viruses Y (PVY) and X (PVX), *Ralstonia solanacearum* (bacterial wilt), *Alternaria solani* (early blight), and two pectinolytic bacteria, *Dickeya* and *Pectobacterium* (blackleg/soft rot). Other pests cause direct damage, such as the Colorado potato beetle *(Leptinotarsa decemlineata*), the peach-potato aphid (*Myzus persicae*), and the potato tuber moth (*Phthorimaea operculella*) (Loebenstein and Gaba 2012; Arora and Sharma 2014; Charkowski et al. 2019). Thus, potato tuber productivity and quality depend on the agrotechnical treatment and the tolerance/sensitivity to multiple stress factors. In addition, several post-harvest factors may affect tuber quality and processing value. For example, long-term storage exposes tubers to diseases, such as soft rot and dry rot caused by *Fusarium* as well as *Colletotrichum coccodes*-generated black dots (Arora and Sharma 2014). Cold storage also induces the accumulation of reducing sugars (RS), a phenomenon described as cold-induced sweetening (CIS) (Isherwood 1973). High levels of RS result in the dark coloration of French fries and chips and in high amounts of neurotoxic and carcinogenic acrylamides after high-temperature processing, which significantly decreases the quality of potato products (Kumar et al. 2004).

During the second half of the twentieth century, substantial efforts were made to improve potato cultivars through conventional breeding. Despite the great amount of work involved, these efforts did not result in replacing older varieties with newer ones more resistant to stresses and capable of producing greater yields. Nowadays, conventional breeding approaches are directed towards enhancing yield, storage, and processing quality, as well as pathogen resistance and abiotic stress tolerance. However, these efforts are challenging as most tuber quality traits, including starch content, CIS-resistance, and nutritional quality (vitamin, carotenoid, and methionine content), are governed by multiple genes (polygenes) (Kloosterman et al. 2010). Thus, the genetic factors associated with each particular trait—including specific allelic variation and precise loci—need to be identified. Conventional breeding, even when assisted by modern molecular techniques, faces numerous challenges due to the narrow gene pool in tetraploid *S. tuberosum*. These challenges, including inbreeding depression, the complexity of genome sequencing, and the unknown complex genetic structure of most of the key traits, impede progress towards potato improvement. One possible solution is to broaden the gene pool of the cultivated potato by using wild potato species in breeding programs, which may provide favorable alleles for traits of interest (mainly resistance to diseases and pests) (Machida-Hirano 2015). However, the introgression of wild potato germplasm into the cultivated potato is both difficult and time-consuming. Ever since the first sequencing of the homozygous double-monoploid (DM 1–3) potato breeding line of the *S. tuberosum* group Andigenum (formerly referred to as cultivar group Phureja) in 2011, studies on candidate genes of special importance for the improvement of potato agronomic and quality traits have become more precise and effective (Xu et al. 2011).

Genetic engineering offers new opportunities to improve potato plants in a relatively short time. It allows for the introduction of genes that can increase their resistance to insects, viral and bacterial diseases, and improve their nutritional value by enhancing protein, vitamin, carotenoid, and lipid content as well as by reducing glycoalkaloid and acrylamide content (Hameed et al. 2018). Additionally, genetically engineered potato plants have attracted interest as a potential source of various products, such as modified starch, lipids, and recombinant proteins, including drugs and vaccines.

To date, several genetically modified (GM) potato cultivars have been commercialized. The first GM varieties, resistant to Colorado potato beetle and PLRV (NewLeaf™, NewLeaf Plus™), were developed by Monsanto^®^ in the late 1990s (Lawson et al. 2001), but their cultivation was abandoned after a few years (reviewed in Hameed et al. 2018). One of the GM potato varieties currently cultivated is the Innate^®^ potato with reduced enzymatic browning (EB) and low acrylamide formation during processing, which was developed by J. R. Simplot Company (Richael 2020). Most GM potato varieties have been engineered using transgene-producing technologies such as transgenesis, RNA interference (RNAi), and cisgenesis (Hameed et al. 2018; Richael 2020).

GM potato varieties are particularly desirable in African sub-Saharan countries. In 2020, potato yields varied between 0.1 t/ha in the Central African Republic and 19.2 t/ha in Zambia, with the average yield of most of the countries in this region being less than10 t/ha—yields far below the average global production (21.8 t/ha) and that of the United States of America (50.8 t/ha) (FAOSTAT 2022). The drivers of this gap include poor seed quality, bacterial wilt, poor soil health, and late blight (Harahagazwe et al. 2018), and it is thus especially important to introduce cultivars resistant to these factors. Recently, the GM Victoria with the three resistance genes (*RB*, *Rpi-blb2*, and *Rpi-vnt1.1*), conferring complete resistance to late blight, has been assessed as promising for cultivation in Africa (Ghislain et al. 2019; Byarugaba et al. 2021). Nevertheless, the widespread use of GM potato cultivars still suffers from low consumer acceptance, despite their great advantages and the lack of substantial differences in agronomic and phenotypic performance compared to conventional cultivars. Thus, new cultivars must simultaneously have a low environmental impact and meet the growing needs of consumers, producers, and the market.

Recently, new breeding techniques (NBTs), including methods based on sequence-specific nucleases (SSNs), have been successfully employed for potato improvement to generate transgene-free plants and tubers for processing. The most commonly used SSNs are transcription activator-like effector nucleases (TALENs) and regularly interspaced palindromic repeats/CRISPR-associated system (CRISPR/Cas) (Hameed et al. 2018). In this review, we discuss successful gene editing using the CRISPR/Cas technology for the improvement of economically important potato traits such as nutritional quality, starch composition, herbicide tolerance, and biotic stress resistance. We also describe the current challenges, opportunities, and limitations concerning CRISPR/Cas technology application in potato.

### Potato functional genomics before CRISPR/Cas

Before the methods based on CRISPR/Cas were widely popularized, many other techniques, including methods to generate loss-of-function mutants, had been developed for functional plant genomics. The application of gene-silencing strategies for reverse genetics and crop improvement in polyploid plants has been particularly challenging. In general, the tools for gene function disruption are based on either RNA targeting (RNA silencing) (Guo et al. 2016) or genome mutagenesis. Both approaches have been adapted for potato plants (Sharma et al. 2022).

The RNA silencing technologies are based on natural and evolutionarily conserved mechanisms in which double-stranded (dsRNA) or hairpin-structured RNA (hrRNA) trigger a reaction cascade ultimately resulting in the degradation of the target mRNA (Guo et al. 2016). Due to their precision and effectiveness, and their applicability in polyploid organisms, RNA silencing technologies, especially those based on RNAi, are widely used in potato research (Sharma et al. 2022). Stable potato modification with RNA expression constructs can be carried out using standard transformation methods. By downregulating endogenous genes, potato lines with new functional features have been produced; these include the high-amylose potato, with decreased expression of genes encoding the starch branching enzymes *SBE*1 and *SBE2* (Andersson et al. 2006), and potato lines with almost completely reduced patatin content in tubers, designed for the production of recombinant human glycoproteins (Kim et al. 2008). Stable RNA silencing is also used to create lines resistant to biotic stress. This approach utilizes host-induced gene silencing (HIGS), in which the RNAi is designed to target plant host susceptibility genes or pathogen genes responsible for causing plant diseases (Hameed et al. 2017; Hussain et al. 2019; De Schutter et al. 2022). The control of potato pathogens and pests can be also supported by spray-induced gene silencing, a highly effective and innovative method in which a dsRNA solution is applied directly to the target plants (Petek et al. 2020).

Among the strategies for introducing changes at the genome level, only a few are available for potato (Oladosu et al. 2016; Sharma et al. 2022). While induced mutagenesis is a popular approach for improving various crop species, it has limited utility in the vegetatively reproducing potato because seeds are the preferred target material for this technique (Oladosu et al. 2016). However, other tissues, especially those suitable for in vitro propagation, such as micro-tubers or callus, have also been used as material for potato mutagenesis (Bradshaw 2021). Another challenging step is detecting the mutagenesis-induced changes, which is mainly due to the tetraploid nature and high allelic variability of potato. These characteristics contribute to the limited applicability of modern methods for precise, large-scale mutation identification, such as targeting induced local lesions in genomes (TILLING) (Rickert et al. 2003; Muth et al. 2008). However, some of these limitations can be circumvented using dihaploid potato. For example, Muth et al. (2008) utilized the TILLING method to identify a series of point mutations in the gene encoding granule-bound starch synthase (*GBSS*) in dihaploid potato lines that were generated from chemically mutagenized seeds. Dihaploid potatoes have also been useful for the production and analysis of insertional mutants generated using transposed Tnt1 elements (Duangpan et al. 2013).

Recently, engineered endonucleases have become a particularly important tool in functional genomics. Zinc-finger nucleases (ZFNs) have rather limited potential as genome editing tools due to their use being complex, time-consuming, and expensive. TALENs, in contrast, are considerably more applicable for several plant species, including potato (Nahirñak et al. 2022). They consist of chimeric proteins, composed of a sequence-specific domain that is fused to a non-specific nuclease (Fok1) and can be easily engineered for targeted genome modification (Christian et al. 2010). The TALEN-edited potato lines generated so far have enabled both the functional analysis of selected genes (Nicolia et al. 2015) and the development of strategies to improve potato commercial properties, such as altered starch quality (Kusano et al. 2016) or enhanced cold storage (Clasen et al. 2016). Compared to RNAi, TALEN-mediated gene editing is a more effective technique as it enables the complete knockout of all alleles. In the case of RNAi, gene silencing can lead to residual protein activity, as was shown for vacuolar invertase Vlnv (Bhaskar et al. 2010; Clasen et al. 2016). Standard plant transformation methods, including protoplast transformation, stable in vitro transformation using *Agrobacterium tumefaciens*, and agroinfiltration, have been successfully used to deliver TALEN constructs to the target plants (Nicolia et al. 2015; Clasen et al. 2016; Kusano et al. 2016; Ma et al. 2017). Many solutions that have been successfully implemented to optimize the various tools of reverse genetics can also be used to improve the efficiency of CRISPR/Cas gene editing in potato.

### Overview of CRISPR/Cas technology

CRISPR/Cas technology is a genome-editing tool that has revolutionized life sciences, including plant research and crop improvement. Compared to ZNFs or TALENs, CRISPR/Cas is cheaper and less laborious, but it is also more prone to off-target effects (Jaganathan et al. 2018; Manghwar et al. 2020). To date, both the most common CRISPR/Cas9 system, and more advanced technologies based on engineered Cas enzymes, have been successfully implemented for gene editing in potato.

The CRISPR/Cas system was first described in bacteria as an adaptive immune system against invading genetic elements, such as phages or plasmids. In 2012–2013, several reports on Cas9 in vitro activity were published (reviewed in Lander 2016), including a paper by Emmanuelle Charpentier, Jennifer Doudna and their colleagues that demonstrated that the CRISPR/Cas system can be used for programmable gene editing (Jinek et al. 2012); this breakthrough discovery earned the two scientists the 2020 Nobel Prize in Chemistry.

CRISPR/Cas9-based gene editing relies on two components: Cas9 endonuclease, which cuts DNA at a specific location, and a single guide RNA (sgRNA), which directs Cas9 to the target site. The sgRNA contains CRISPR RNA (crRNA), a 20-nt sequence complementary to the target sequence (called the protospacer), and trans-activating crRNA (tracrRNA), which recruits the endonuclease. The Cas9 from *Streptococcus pyogenes* (SpCas9) is most widely used for gene editing in plants. It belongs to the class 2 type II CRISPR system and contains two nuclease domains: HNH and RuvC, which cut the complementary and non-complementary strands of the target DNA, respectively. A Cas9-mediated DNA cleavage requires an NGG protospacer adjacent motif (PAM) directly downstream of the target sequence (Puchta 2017). The double-strand breaks (DSBs), introduced by the Cas9 3–4 bp upstream of PAM, are repaired either through nonhomologous end-joining (NHEJ) or homologous recombination (HR), also known as homology-directed repair (HDR) (Das Dangol et al. 2019). The NHEJ is the dominant repair mechanism in plant somatic cells; as an error-prone process, it generates small insertions or deletions, resulting in the knockout of the target gene. The HR pathway can be used for the integration of a supplied donor DNA at the DSBs site, but its use is limited due to low efficiencies (Steinert et al. 2016). Nevertheless, many reports have shown that it is possible to enhance HR efficiency in plants by implementing different approaches, such as modulating environmental factors, suppressing the NHEJ pathway or using geminivirus-based replicons (GVRs) to increase the amounts of the donor template (Chen et al. 2022).

The number of potential target sites for SpCas9 in a gene of interest can be limited by the NGG PAM requirement. To tackle this problem, Cas9 orthologs from other bacterial species, recognizing alternative PAMs, have been used for gene editing in different plant species, including Arabidopsis (*Arabidopsis thaliana)*, rice (*Oryza sativa* L.), and tobacco (*Nicotiana tabacum* L.) (Wada et al. 2022). In addition, SpCas9 has been engineered to recognize other PAMs, including NGA (Cas9-VQR), NG (Cas9-NG), and NG/GAA/GAT (xCas9) (reviewed in Zhang et al. 2019). The Cas9 variants with a relaxed PAM requirement have been primarily tested in Arabidopsis and rice (Wada et al. 2022).

Modifications of Cas9 also targeted its endonuclease activity. Point mutations in HNH (H840A) or RuvC (D10A) domains enabled to create nCas9 nickase, which generates a nick, instead of a DSB, at the target site. The simultaneous introduction of both mutations leads to obtaining a catalytically dead Cas9 (dCas9) (Zhang et al. 2019). Such an inactive Cas9 retains its DNA-binding activity guided by the sgRNA and can therefore be used for the regulation of gene transcription through its fusion to various transcriptional regulators (reviewed in Moradpour and Abdulah 2020).

Generation of Cas9 mutants lacking the capacity to introduce DSBs facilitated the development of more precise genome editing technologies: base (BE) and prime editing (PE). Base editors, which are fusions of nCas9 or dCas9 and a nucleotide base deaminase, introduce point mutations in the target DNA sequence. The cytosine base editor (CBE) induces C-to-T substitution, whereas the adenine base editor (ABE) catalyzes A-to-G conversions (Huang and Puchta 2021). Currently, several versions of both CBE and ABE are available, and some have already been applied in a large variety of crops to modify genes involved in properties such as herbicide resistance and nitrogen use efficiency (Li et al. 2021; Zhu and Zhu 2022). Recently, a cytosine transversion base editor (CGBE) capable of introducing C-to-G change and dual base editors with the ability to simultaneously convert C to T and A to G have been developed (Li et al. 2021). The prime editing strategy (the so-called “search-and-replace technology”) allows the introduction of a designed edit at a specific locus. The method uses nCas9 fused to an engineered reverse transcriptase (RT) and a prime editing guide RNA (pegRNA) containing sgRNA, a primer binding site (PBS), and an edit-encoding extension (Butler et al. 2015). The PAM-containing DNA strand is nicked by nCas9, and the resulting 3ʹ flap hybridizes with the PBS. Subsequently, RT uses the edit as a template and generates the 3ʹ flap containing the copy of the edited sequence. The 5ʹ flap is preferentially removed by endonucleases, and the 3ʹ flap is ligated to the DNA strand. After DNA repair, the edited sequence is permanently introduced into the genome (Anzalone et al. 2019; Wada et al. 2020). The first attempts to apply PE to plants have shown that the editing efficiency is lower compared to that in mammalian cells, and therefore, further optimization is necessary (Hassan et al. 2021).

The exploration of other types of the class 2 CRISPR/Cas system has led to the discovery of Cas enzymes with useful properties. One of these is Cas12a (formerly known as Cpf1), which enables the editing of AT-rich genomes (reviewed in Bandyopadhyay et al. 2020). In contrast to Cas9, Cas12a requires only a single crRNA and recognizes T-rich PAMs located upstream of the target sequence. In addition, it generates DSBs with staggered ends, which can result in larger deletions. Due to its RNase activity, Cas12a can specifically process crRNA arrays and, therefore, represents a powerful tool for multiplex genome editing (Zhang et al. 2019, 2021). Among the Cas12a variants, endonucleases from *Francisella novicida* (FnCas12a), *Lachnospiraceae bacterium* (LbCas12a), and *Acidaminococcus* sp. (AsCas12a) are most frequently used for plants (Bandyopadhyay et al. 2020). Interestingly, their activity is temperature-dependent, which can be a limiting factor for plant genome editing (Malzahn et al. 2019). Consequently, the temperature tolerance of Cas12a variants was improved by genetic engineering, which enabled more efficient gene editing in plants grown at lower temperatures (Schindele and Puchta 2020).

Another type of Cas protein, CRISPR-associated protein 13 (Cas13), exclusively recognizes and cuts single-stranded RNA. The cleavage is mediated by two eukaryote and prokaryote nucleotide-binding domains (HEPN) (Wolter and Puchta 2018). The CRISPR/Cas13 system is used for RNA knockdown (Wada et al. 2022) and to enhance plant immunity against RNA viruses (Mahas et al. 2019). Deactivation of HEPN domains converts Cas13 into the programmable RNA-binding protein (dCas13), which, after fusion with fluorescent tags, can be employed for the imaging of RNA in live cells (Abudayyeh et al. 2017). Similar to dCas9, the catalytically inactive Cas13 was fused with the deaminase domain of adenosine deaminase acting on RNA (ADAR2), and used for precise transcript editing (Cox et al. 2017).

Although the development of CRISPR/Cas technology in plants is based on the most popular research models, Arabidopsis and rice, there is also a growing body of research aiming to apply these approaches to species with more complex genetics. Potato is one of these as its complex biology poses a challenge for optimizing the latest gene-editing methods

## Potato as a target plant for CRISPR/Cas genome editing technology

**Fig. 1:**
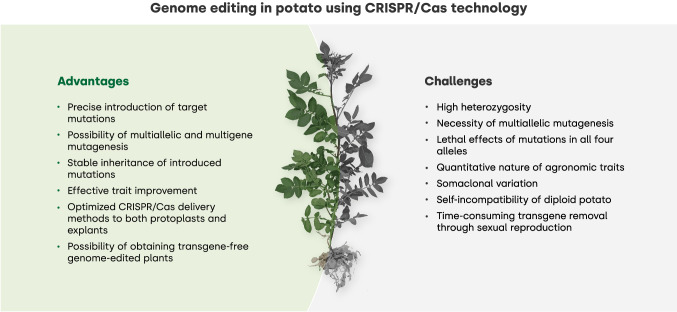
Advantages and challenges of CRISPR/Cas-mediated improvement of tetra- and diploid potato. The optimization of CRISPR/Cas approaches in potato has enabled not only the precise introduction of mutations but also the efficient editing of multiple genes and alleles. Introduced mutations can be inherited and often result in the improvement of the desired trait. The availability of different delivery methods of CRISPR/Cas components to both protoplast and explants allows for obtaining transgene-free plants. However, gene editing in potato is still challenging due to the large genome size and the specific biology of the species. The tetraploid genome with high heterozygosity creates the possibility of interactions between alleles and imposes the necessity of multiallelic mutagenesis which may result in a lethal effect. The quantitative nature of the agronomic traits makes it difficult to choose specific candidate genes underlying the trait. Moreover, potato plants regenerated from explants or protoplasts, cultured in vitro, are prone to somaclonal variation manifested as polyploidization, methylation in off-target genes and morphological abnormalities. Some of the difficulties in gene editing in tetraploid potato can be overcome by using a diploid potato. However, the self-incompatibility of a diploid potato limits the development of the inbred lines and thus hinders the introduction of desired mutations into the germplasm. Finally, for lines stably transformed with CRISPR/Cas constructs, the transgene removal is time-consuming as it requires subsequent sexual crossings

Potato, due to its critical role in feeding the world and its wide use as raw material for the food-processing industry, is an important target for genetic improvement. The application of biotechnological techniques such as genetic engineering and genome editing significantly reduces the time required to introduce or modify a targeted gene, particular allele, or sequence in potato from years to months, and it does so without reshaping allelic combinations (Barrell et al. 2013). Thus, the application of CRISPR/Cas technology to improve potato, especially their quality traits, is a promising approach with numerous advantages (Fig. 1, left panel).

Genome editing must face the obstacles of complex potato genetics (Fig. 1, right panel). The cultivated tetraploid (2n = 4*x* = 48) potato *S*. *tuberosum* subs. *tuberosum* has four sets of chromosomes and tetrasomic inheritance (Watanabe 2015). Diversity of alleles at a locus increases heterozygosity and results in multiallelic interactions and induces intra-locus and epistatic interactions (Manrique-Carpintero et al. 2018). High heterozygosity is also problematic for potato transformation/genome editing; it impedes the effective removal of a transgene through allelic segregation during sexual reproduction and results in a loss of optimal genetic integrity that is superior for desired traits (Barrell et al. 2013). High polymorphism rates in multiple alleles, which, in the case of the *S-locus RNase* (*S-RNase*) gene, can result in up to 94% amino acid sequence variation, present an obstacle in the design of the four allele-targeting sgRNAs used for CRISPR/Cas-mediated editing. However, this can be circumvented by designing sgRNAs that target regions encoding conserved domains in the protein, common for all alleles (Ye et al. 2018; Enciso-Rodriguez et al. 2019; see the section *Generation of self-compatible diploid potato*).

Most agronomic traits of potato are of quantitative nature. This means they are governed by multiple genetic factors including regulatory genes, microRNA, preferential and multiple alleles (e.g., starch and chip color, potato tuber greening, tuber shape, specific gravity) (Schäfer-Pregl et al. 1998; Śliwka et al. 2016; Sołtys-Kalina et al. 2020b, a; Plich et al. 2020; Park et al. 2021). The quantitative nature of interactions at the genome level creates the risk that genetic manipulation of one gene/allele will not change the phenotype and that the desired trait will not be achieved. For example, Clasen et al. (2016) showed that the number of mutated alleles of the *VInv* gene, responsible for RS accumulation, was correlated with the RS content; only lines with knockout mutations in all four alleles were characterized by the RS content below the limit of detection. However, another study demonstrated that the knockout of all four alleles may become lethal, as in the case of the homozygous mutation in hydroxy-2-methyl-2-(E)-butenyl 4-diphosphate synthase gene in the CRISPR/Cas9-edited cv. Désirée (Kieu et al. 2021). Another problem associated with generating full allelic mutations in potato is the occurrence of numerous developmental abnormalities in the modified plants. These can include impaired root formation (lines with *PHYTOENE DESATURASE* (*PDS*) gene mutation; Bánfalvi et al. 2020) and pleiotropic phenotypes with altered morphology, such as growth inhibition, long and thin stems, and leaf necrosis, which were observed when knocking out the susceptibility gene (*S*-gene) encoding a cyclic nucleotide-gated ion channel (*StDND1*) (Kieu et al. 2021). All these abnormalities made the *Stdnd1* mutant lines unsuitable for use in agriculture (Kieu et al. 2021).

Genome editing, which involves the transformation of protoplasts or explants, requires the regeneration of a whole plant in in vitro culture conditions. Plants after regeneration may present phenotypic and genotypic differences compared to those from which they were regenerated; this phenomenon is called somaclonal variation (Bordallo et al. 2004; Miguel and Marum 2011). Somaclonal variation can occur through DNA methylation, single nucleotide mutations, or changes in chromosome number and structure, and it may affect the introgression of transgenes and the governance of targeted traits since it causes various undesirable changes in off-target genes (Karp and Bright 1985; Fossi et al. 2019). As a result, the desired phenotype is not obtained despite successful gene incorporation/genome editing. Potato is prone to somaclonal variation, with additional transformation increasing its frequency (Karp and Bright 1985). Regenerated plants may possess morphological changes, including altered shape, leaf color, deformated tubers and flowers, and may exhibit a dwarf phenotype (Rietveld et al. 1993; Thieme and Griess 2005). Fossi et al. (2019) compared potato plants regenerated from the cv. Désirée via stem explants (control), protoplasts, and stem explants after *Agrobacterium*-mediated transformation. Among plants regenerated from the protoplasts, both normal and abnormal phenotypes were observed. Although the plants exhibited phenotypes resembling the starting clone, the regeneration resulted in high genome instability and included multiple changes in chromosomes, deletions, and duplications. Plants regenerated from explants transformed with *Agrobacterium* displayed similar genomic changes as those regenerated from protoplasts, including large-scale chromosome deletions, duplications, and trisomy (Fossi et al. 2019). Such genomic instability may affect the phenotypes of plants regenerated after CRISPR/Cas delivery (for more information see the section *CRISPR/Cas delivery methods in potato*). Moreover, DNA breaks in plants regenerated from protoplasts and *Agrobacterium-*transformed explants seem to occur at specific sites in the genome, suggesting a non-randomness that may result from the structure of the potato genome itself (Fossi et al. 2019). Chromosome doubling as a consequence of somaclonal variation was also noted in the CRISPR/Cas-edited diploid potato after knockout of the self-incompatibility gene (Ye et al. 2018; Enciso-Rodriguez et al. 2019). In potato, as a vegetatively propagated crop, the occurrence of genomic changes and their accumulation in successive vegetative reproductions significantly affects agronomic performance, and this effect should be considered when editing the genome (Fossi et al. 2019).

Despite challenges related to potato genome structure and phenotype/genotype variation during plant regeneration after genome editing, CRISPR/Cas technology has great potential for effectively implementing potato modifications, given that the most successful genetically manipulated traits in potato have been achieved through gene silencing/knockout.

## Applications of CRISPR/Cas in potato

**Fig. 2:**
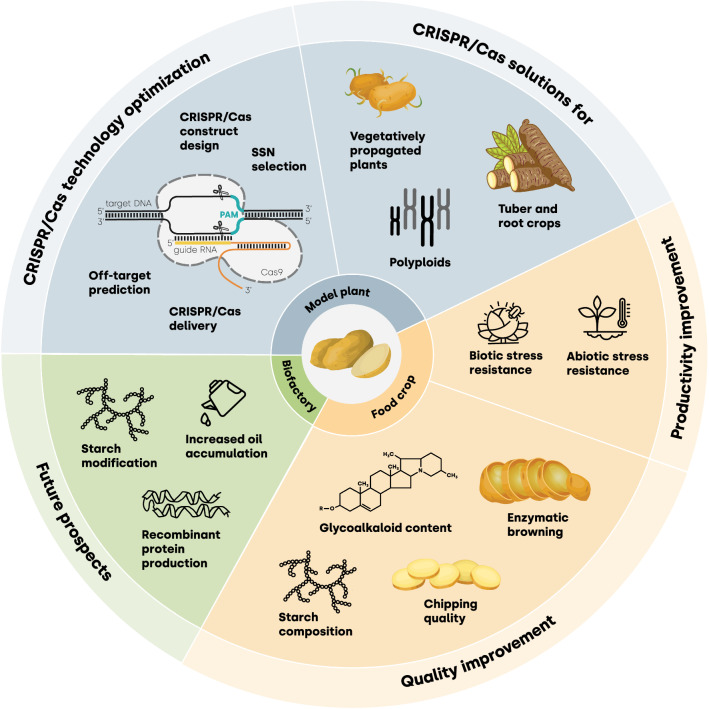
Diversity of CRISPR/Cas applications in potato research. The current research work on the development of CRISPR/Cas techniques in potato focuses on three areas (clockwise from the top): potato as a Model plant for CRISPR/Cas efficiency improvement (CRISPR/Cas technology optimization) and for the development of new CRISPR/Cas tools useful for other tuber crops, polyploids, and vegetatively reproducing plants (CRISPR/Cas solutions for); potato as a Food crop comprising all experimental approaches aimed at enhancing tuber productivity (Productivity improvement) and quality (Quality improvement) in potato cultivars for food and industrial purposes; and Future prospects covering possible research directions towards using potato for the production of tailor-made starch, lipids, and recombinant proteins

The use of CRISPR/Cas technology for potato genome editing has, so far, followed two main research paths (Fig. 2). The first path has focused on technical aspects and has involved testing new ideas for improving CRISPR/Cas procedures in potato (Wang et al. 2015) and implementing innovations that have been successfully established in other organisms (Zong et al. 2018; Perroud et al. 2022). The resulting solutions can ideally be further adopted for genome editing in other tuber and polyploid crops (Fig. 2, Model plant). The second path has employed CRISPR/Cas techniques to create or improve phenotypic features and has often generated genome-edited lines with the potential for commercialization (Andersson et al. 2017; Kusano et al. 2018; Carlsen et al. 2022) (Fig. 2, Food crop).

### Potato as a model plant

Due to numerous applications and interesting biology, potato cultivars are often selected as research models in both basic and applied research (Nahirñak et al. 2022). The primary factors that make potato an attractive research model include the simplicity of potato cultivation both in the field and under laboratory conditions, including in vitro, and the availability of optimized protocols for protoplast isolation and tissue culture of roots and tubers, thereby enabling the implementation of modern gene/genetic technologies (for review, see Hameed et al. 2018; Nahirñak et al. 2022; Tiwari et al. 2022). It should be noted that potato is one of the few species for which effective in vitro regeneration procedures from a single protoplast to a fully organized plant have been developed (Mao et al. 2019; Rather et al. 2022).

As the main representatives of tuber crops and vegetatively reproducing polyploids, potatoes are frequently selected to test the usefulness of the latest NBTs aimed at improving plant quality and productivity (Hameed et al. 2018; Tussipkan and Manabayeva 2021; Sharma et al. 2022). Among the potato tetraploid cultivars, Désirée is most commonly used in studies involving the application of CRISPR/Cas technologies (Nahirñak et al. 2022; Tiwari et al. 2022; Sharma et al. 2022). For this cultivar, various research tools are currently available, ranging from numerous established protocols for genetic manipulation to large amounts of data for omics studies and the recently released SNP map (Sevestre et al. 2020; Nahirñak et al. 2022). However, Désirée is not an ideal candidate for testing genome editing approaches; the analyses of quantitative traits, the selection of candidate genes underlying these traits, and the screening of mutated genes are more complex and time-consuming in tetraploid cultivars (Nahirñak et al. 2022; Sharma et al. 2022). Therefore, potatoes with a low ploidy level, such as the wild species *S. chacoense*, and with reduced ploidy, such as artificially generated dihaploids (DHs), are good candidates for reverse genetics studies and testing CRISPR/Cas techniques (Brigneti et al. 2004; Duangpan et al. 2013; Butler et al. 2015, 2016). The DH lines can also be the starting material for obtaining homozygous tetraploid mutant lines, although this procedure requires dealing with the frequently occurring phenomenon of self-incompatibility (Ye et al. 2018; Enciso-Rodriguez et al. 2019; see also the section *Generation of self-compatible diploid potato*).

To date, most of the available reports on CRISPR/Cas technologies applied in potato have been focused on the optimization of the techniques previously tested in other species (e.g., Wang et al. 2015; Andersson et al. 2017; Zong et al. 2018; Perroud et al. 2022). Although the CRISPR-based potato editing techniques require improvement, particularly with regards to the unique obstacles associated with the potato, the solutions developed so far may be useful for other tuber and polyploid plants.

#### Marker (Reporter) genes for CRISPR/Cas testing in potato

In plant systems, endogenous genes that give a uniform and easily detectable phenotypic effect are frequently selected for testing new genome editing techniques. Two of them, *ACETOLACTATE SYNTHASE* (*ALS*) and *PHYTOENE DESATURASE* (*PDS*), have also proven to be useful for optimizing CRISPR/Cas approaches in potato (Faivre-Rampant et al. 2004; Nekrasov et al. 2013; Butler et al. 2015; Veillet et al. 2019b; Bánfalvi et al. 2020).

The enzyme ALS catalyzes the first step of the synthesis of the branched-chain amino acids valine, leucine, and isoleucine. Since ALS is essential in the plant cell, it is most commonly used to test gene editing techniques or approaches that introduce point mutations in *ALS* that do not lead to a complete loss of enzyme activity. The native enzyme is competitively inhibited by structurally different herbicides, such as sulfonylureas, triazolopyrimidine sulfonamides, or pyrimidinylsalicylates, whereas many of its mutant variants show much lower affinity to these substances (Shimizu et al. 2002; Ray et al. 2004; Yu et al. 2010). Various *ALS* mutations resulting in increased herbicide resistance have, so far, been identified in numerous plant species. For example, mutation of the Proline-197 (Pro-197) residue (aa number standardized to the sequence of Arabidopsis, corresponding to Pro-186 in potato StALS1) results in chlorsulfuron (sulfonylurea compound) resistance and is one of the most common mutations (Yu et al. 2010). Introducing such targeted mutations within *ALS* using CRISPR/Cas technology may allow for the generation of herbicide-resistant plants for commercial use, including tetraploid potato (Hofvander et al. 2022). In potato research, *StALS1* was a target for some of the pioneering work on CRISPR/Cas-mediated gene editing in both diploid and tetraploid cultivars as well as for testing the applicability of the novel, precise genome editing technologies, BE and PE (Butler et al. 2015, 2016; Zong et al. 2018; Veillet et al. 2019a; Perroud et al. 2022).

In plants, PDS is involved in carotenoid synthesis (Nekrasov et al. 2013). The application of *PDS* as a marker for testing the knockout efficiency in potato diploid and tetraploid model lines has previously been demonstrated (Faivre-Rampant et al. 2004; Brigneti et al. 2004). The knockout of all *PDS* alleles results in total plant albinism, which is easy to detect visually, even in the early stages of stem regeneration (Pan et al. 2016; Chen et al. 2018). The systemic silencing of *PDS* in potato is detectable in the foliar tissues and in tubers, including the in vitro-generated microtubers (Faivre-Rampant et al. 2004; Brigneti et al. 2004). For CRISPR/Cas gene editing in potato, *PDS* was used to test the efficiency of newly designed vectors or post-transformation selection methods (Bánfalvi et al. 2020). Regenerated plants can be preliminarily selected based on their phenotype for the further confirmation of tetra-allelic mutations at the molecular level.

Additionally, the enzyme GBSS is also commonly used as an endogenous reporter in the cultivated tetraploid potato, as it is encoded by a single locus with four alleles (*StGBSS*, also designated as *StGBSSI*) (Andersson et al. 2017). Full allelic mutation in *StGBSS* results in easily detectable changes in the starch structure (high-amylopectin potato; waxy potato) (Nicolia et al. 2015; Andersson et al. 2017; Kusano et al. 2018; Veillet et al. 2019a), and thus the knockout of *StGBSS* can be detected using quantitative and qualitative methods for starch analysis in tubers growing in vivo and in vitro (Andersson et al. 2017; Veillet et al. 2019a). Due to the utility potential of the GBSS-deficient potato, these phenotypic effects will be discussed later in this review (see the section *Modification of starch composition*).

The marker genes described above fully meet the necessary conditions for their use as reporter/selection targets in potato: (1) enabling the monitoring of the effectiveness of new techniques for genome editing; (2) facilitating the detection of mutated events (e.g., co-transformation with the CRISPR/Cas constructs targeting *ALS* and selected endogenous gene/genes); (3) enabling the selection of genome-edited events; and (4) supporting the generation of non-transgenic genome-edited plant lines (see the section *Developing strategies for the generation of non-transgenic genome-edited potato*).

#### CRISPR/Cas delivery methods in potato

The method used to deliver CRISPR/Cas components to target cells directly impacts the efficiency of the gene editing (Khatodia et al. 2016); similar effect has also been observed in potato (Das Dangol et al. 2019; González et al. 2021). Among the numerous transformation methods proposed to generate transgenic potato, the effective delivery of CRISPR/Cas components has been confirmed so far only for *Agrobacterium*-mediated transformation and for protoplast transfection (Tiwari et al. 2022). In the case of protoplasts, efficient target mutagenesis can be achieved by both transient expression of DNA constructs containing CRISPR/Cas genes and a direct application of active Cas/sgRNA nucleoprotein complexes (RNPs) (Andersson et al. 2017, 2018; Zong et al. 2018; Veillet et al. 2019a; González et al. 2021; Carlsen et al. 2022).

Independent studies have shown that both stable and unstable agrotransformation methods can be used for CRISPR/Cas delivery to target plant tissues (e.g., Butler et al. 2015; Andersson et al. 2017; Veillet et al. 2019b; Bánfalvi et al. 2020). The stable integration of the CRISPR/Cas transgene into the target genome increases the chances of generating full allelic mutations in regenerated potato lines, which is presumably associated with a higher and more stable Cas nuclease activity compared to the transient transformation (Bánfalvi et al. 2020). Moreover, the mutations detected in potato lines with the stable integration of *Cas* and *sgRNA* can be much more extensive. For example, Bánfalvi et al. (2020) demonstrated that Désirée mutants stably transformed using the CRISPR/Cas plasmid targeting *PDS* showed large deletions (> 100 bp), whereas the lines without the integrated construct possessed substitutions and deletions of only a few bp. The protocols proposed for stable potato transformation with the CRISPR/Cas components are optimized based on specific factors including the selection of effective bacterial strains, binary vectors, and target tissues (Wang et al. 2015; Das Dangol et al. 2019; Nahirñak et al. 2022). The proper selection of these factors is important to improve the efficiency of genome editing procedures. Various potato cultivars show differences in susceptibility to in vitro cultivation and transformation, which also affects the effectiveness of genome editing techniques (Nahirñak et al. 2022).

While there are numerous possibilities and advantages of stable *Agrobacterium*-mediated transformation for CRISPR/Cas delivery, there may also be undesirable effects. These effects, which may interfere with the interpretation of the phenotypes observed in the edited lines, include transgene silencing and destabilization as well as effects related to the random integration of T-DNA into the host genome (Gelvin 2017). However, some of these effects can be circumvented by using transient transformation methods. These methods do not involve the integration of exogenous DNA into the target genome and thus allow for temporary CRISPR/Cas gene expression (González et al. 2020). Unstable transformation strategies are of particular importance in genome editing as they allow for the generation of non-transgenic plants (see the section *Developing strategies for the generation of non-transgenic genome-edited potato*).

#### Optimization of CRISPR/Cas approaches in potato

In the first reports on CRISPR/Cas editing in potato, it was emphasized that this technology could find application both as a tool to study the functions, regulation mechanisms, and interactions of genes and to improve the potato traits important for the processing industry (Wang et al. 2015; Butler et al. 2015, 2016). The success in potato editing was based on approaches that addressed the demanding biology of this species. Most of them combined methods tested previously in other plants with solutions unique for potato (Table 1). For example, Wang et al. (2015) cloned a native promoter for potato U6 RNAs (*StU6p*) to drive the sgRNA targeting endogenous *StIAA* (encoding Aux/IAA protein). They also used the diploid breeding line as a research model, which increased the likelihood of the induction of full-allelic mutants. This combination enabled the generation of biallelic and monoallelic homozygous mutant lines (Wang et al. 2015). Other studies have also demonstrated the usefulness of native RNA promoters isolated from potato (Andersson et al. 2017; Veillet et al. 2019a; Johansen et al. 2019). For example, Johansen et al. (2019) compared the activities of four *U6* promoters isolated from cvs. Désirée and Wotan with the commonly used *U6-1* promoter from Arabidopsis (*AtU6-1p*). Replacement of the *AtU6-1p* with endogenous potato *StU6* promoters resulted in up to a ninefold increase in the editing efficiency of the target *StGBSS* (Johansen et al. 2019).

**Table 1:** Strategies for adapting the CRISPR/Cas system to potato genome editing

Strategies		Benefits	Obstacles	References
*Solutions specific for potato*				
Selecting RNA promoters from potato	*StU6* and *StU3* promoters	Increased sgRNA expression. Increased editing efficiency	Requires searching for new promoter sequences in databases, their synthesis and insertion into appropriate plasmids, and preliminary testing the activity of new constructs	Wang et al. 2015, Anderson et al. 2017, Veillet et al. 2019b, Johansen et al. 2019
Selecting appropriate target plants	Dihaploids or diploid genotypes/species	Less complicated design of sgRNAs targeting two alleles. Higher frequency of full allelic mutants compared to tetraploid genotypes. Less time-consuming and easier screening of diploid transformants than tetraploid ones	Frequent self-incompatibility. Requires optimization of plant regeneration protocols for genome-edited explants/protoplasts Low commercialization potential	Butler et al. 2015, Wang et al. 2015, Butler et al. 2016, Ye et al. 2018, Enciso-Rodriguez et al. 2019, Carlsen et al. 2022
	Model cultivars commonly used for genetic modification, e.g., Désirée	Availability of numerous experimental protocols including optimized procedures for obtaining stable and transient transgene expression	More complicated design of sgRNAs targeting all four alleles. Lower frequency of full (tetra) allelic mutants compared to diploid genotypes. More difficult and time-consuming screening of tetraploid transformants than diploid ones. Low commercialization potential	Wang et al. 2015, Veillet et al. 2019a, Banfalvi et al. 2020, Gonzales et al. 2021
	Commercial potato cultivars, e.g., Rywal, Kuras, Sayaka, Mayqeen, Atlantic	High commercialization potential. Possibility to select a potato cultivar with a set of traits most suitable as a background for planned genomic editions. Possibility to use protocols developed for dihaploids or diploid genotypes/species or the cv. Désirée	More complicated design of sgRNAs targeting all four alleles. Lower frequency of full (tetra) allelic mutants compared to diploid genotypes. More difficult and time-consuming screening of tetraploid transformants than diploid ones. Adaptation of protocols developed for dihaploids or diploid genotypes/species or the cv. Désirée for other potato cultivars often requires additional optimization procedures	Andersson et al. 2017, Kusano et al. 2018, Nakayasu et al. 2018, Zheng et al. 2021, Lukan et al. 2022, Carlsen et al. 2022, Toinga-Villafuerte et al. 2022
*Solutions successfully adapted from other plants*				
Enhancing *Cas* expression	Constitutive promotors: *CaMV 35S* promoter, duplicated *CaMV 35S* promoter, ubiquitin 4–2 promoter from parsley	High expression of CRISPR/Cas genes. Easy availability of ready-made constructs. Confirmed functionality in various plant expression systems	Limited possibility of controlling effects of a constitutively active Cas, such as increased frequency of off-target mutations. Possible occurrence of mosaic effects in generated mutants due to heterogeneous expression from *35S* promoter	Veillet et al. 2019a, Veillet et al. 2019b, Zong et al. 2018, Fauser et al. 2014, Kusano et al. 2018
	Strong terminators: *CaMV 35S* terminator, nopaline synthase terminator from *Agrobacterium tumefaciens*; pea3A terminator from pea			
	Translational enhancers from rice: dMac3 from 5'UTR of *OsMac3* and ADH from 5'UTR of *OsADH*	Increased *Cas* translation. Enhanced Cas activity	Increased probability of off-target mutations due to higher Cas activity	Kusano et al. 2018, Takeuchi et al. 2021
	*Cas9* (with NLS) codon-optimized for plants: Arabidopsis *(AtCas9),* rice *(OsCas9),* and dicotyledonous plants	Enhanced Cas activity in various species, including potato	Expression of *Cas* gene codon-optimized for unrelated genomes may be reduced in potato	Butler et al. 2015, Wang et al. 2015, Butler et al. 2016, Kusano et al. 2018, Veillet et al. 2019a, Veillet et al. 2019b
Using engineered variants of Cas9	nCas9/dCas9 fusions for precise editing	Precise edition of genes without inducing DSBs. Programmable conversion of single nucleotides. Precise replacement of DNA fragments	Further optimization in potato needed. More complex design of BE/PE systems than for the standard CRISPR/Cas technology	Zong et. al. 2018, Veillet et al. 2019a, Veillet et al. 2019b, Perroud et al. 2022
	Cas9 recognizing non-canonical PAMs (SpCas9-NG)	Increased targeting range of Cas9	Further optimization in potato needed	Veillet et al. 2020b
Designing multiple sgRNA sequences	Two or more sgRNAs	Targeting several sites in a target gene. Increased effectiveness of introducing targeted mutations and likelihood of tetra-allelic mutations	More complex constructs. More potential off-targets	Andersson et al. 2017, Kusano et al. 2018, Carlsen et al. 2022, Lukan et al. 2022
Testing plant RNA promoter/-s	Arabidopsis U6 promoter (*AtU6*)	Effective and universal for dicotyledonous plants	Less effective than native potato promoters	Kusano et al. 2018, Banfalvi et al. 2020, Gonzales et al. 2021, Perroud et al. 2022
Using geminivirus replicons (GVRs)	Elements from the bean yellow dwarf virus (BeYDV)	Improved frequency of HR-generated mutations	No clear evidence that the GVR-based CRISPR/Cas vectors are more effective in potato than those containing the standard T-DNA backbone	Butler et al. 2015, Butler et al. 2016
Optimizing CRISPR/Cas delivery method	*Agrobacterium*-mediated stable transformation	Possible generation of tetra-allelic mutants. Possible induction of large fragment deletions/insertions	Frequently occurring mosaic effects and somaclonal variations in edited plant tissues. Not recommended for generating non-transgenic genome-edited potatoes	Kusano et al. 2018, Veillet et al. 2019a, Veillet et al. 2019b, Banfalvi et al. 2020, Perroud et al. 2022, Lukan et al. 2022
	*Agrobacterium*-mediated transient transformation	Possibility to generate non-transgenic genome-edited lines	Lower efficiency than in stable transformation. Very low probability to induce large fragment deletion/insertion	Veillet et al.2019a, Banfalvi et al. 2020, Perroud et al. 2022
	Protoplast transfection with plasmids carrying CRISPR/Cas components	High frequency of tetra-allelic mutations. Possibility to regenerate whole plants. Possibility to generate non-transgenic genome-edited lines	Plasmid fragments may be inserted into a target genome. High risk of somaclonal changes in regenerated plants	Andersson et al. 2017, Zong et al. 2018, Veillet et al. 2019b, Carlsen et al. 2022, Lukan et al. 2022
	Protoplast transfection with RNPs	Possible generation of tetra-allelic mutations. Possibility to regenerate whole plants. Possibility to generate non-transgenic genome-edited lines	High risk of somaclonal changes in regenerated plants	Andersson et al. 2017, Johansen et al. 2019, González et al. 2021, Zhao et al. 2021, Carlsen et al. 2022

*BE* base editing, *DSBs* double-strand breaks, *NLS* nuclear localization signal, *PAM* protospacer adjacent motif, *PE* prime editing, *RNPs* Cas/sgRNA nucleoprotein complexes

To develop another CRISPR/Cas genome editing strategy in potato, Butler et al. (2015, 2016) tested GVR-based vectors. The advantages of GVRs as a delivery system for SSNs have been shown using tobacco as an experimental model. This method has resulted in increased gene targeting efficiency of one to two orders of magnitude higher than that of conventional *Agrobacterium*-mediated T-DNA delivery, an effect that has been replicated in other plant expression systems, including tomato (*Solanum lycopersicum* L.) (Baltes et al. 2014; Dahan-Meir et al. 2018; Bhattacharjee and Hallan 2022). The effectiveness of GVRs as CRISPR/Cas delivery systems has also been tested using diploid breeding line and Désirée cultivars (Butler et al. 2015, 2016). Butler et al. (2015) showed that GVR-mediated delivery of one of the SSNs, Cas9 or TALENs, targeting the potato *StALS* gene, combined with the repair templates designed to incorporate point mutations within the *ALS* locus can be used to introduce HR-generated mutations (Butler et al. 2015). Although vectors based on GVRs are considered a promising system for plant genome editing (Baltes et al. 2014; Dahan-Meir et al. 2018), the results obtained so far using potato as a research model indicate the need for further optimization.

Another important research direction for optimizing CRISPR/Cas in potato has focused on developing methods that enable the induction of changes for all alleles of the targeted gene (Andersson et al. 2017; Nicolia et al. 2021). These methods may successfully replace time-consuming crosses of potato lines with an introduced mutation, which have been used to achieve full allelic mutations. The possibility of avoiding subsequent crosses also allows for reducing the negative effects that may occur as a result of inbreeding depression. The successful generation of tetra-allelic mutants using CRISPR/Cas constructs that can simultaneously target multiple sites on the selected gene has been shown in stably and transiently transformed potato cultivars (Andersson et al. 2017; Kusano et al. 2018; Veillet et al. 2019b; Chauvin et al. 2021). For example, Andersson et al. (2017) used transient expression of CRISPR/Cas9 genes in protoplasts isolated from the tetraploid cv. Kuras to induce mutations in *StGBSS*. This approach resulted in mutations in all four alleles of *StGBSS* in up to 2% of regenerated lines (Andersson et al. 2017).

Other studies have focused on improving Cas activity and the use of multiple sgRNAs. Kusano et al. (2018) used the tetraploid potato cv. Sayaka to show that a significant increase in CRISPR/Cas9 editing efficiency can be achieved by using the translational enhancer dMac3 from 5ʹUTR of rice *OsMac3*, the rice-optimized *Cas9* gene under the duplicated *35S* promoter, and three sgRNAs targeting multiple sites in the *StGBSS* (Mikami et al. 2015; Kusano et al. 2018). More than 25% of plants regenerated after stable *Agrobacterium*-mediated transformation showed the presence of four allelic mutations in the *GBSS*, whereas the editing effectivity was significantly lower (less than 10% of full allelic mutants) when the vector variants with only two sgRNAs and/or without the dMac3 were used (Kusano et al. 2018). In the most recently published study, Lukan et al. (2022) demonstrated the effectiveness of using CRISPR/Cas9 to modulate miRNA expression in two potato cultivars, Désirée and Rywal, through the use of dual sgRNA constructs designed for targeting both 5ʹ and 3ʹ ends of three selected MIR genes (Lukan et al. 2022).

#### Testing the latest CRISPR/Cas technology

The innovative techniques for precise genome editing, such as PE and BE, were tested in various plants using both stable and unstable transformation methods (Nahirñak et al. 2022). Zong et al. (2018) demonstrated the first use of BE in potato. This work used a novel base editor fusion protein, A3A-PBE, containing the Cas9 (D10A) nickase and the human cytidine deaminase APOBEC3A. The A3A-PBE-coding sequence was codon-optimized for expression in cereal plants and was tested in several model plants including wheat (*Triticum aestivum* L.), rice, and potato. In contrast to the previous version of the plant base editor (Zong et al. 2017), which was based on the rat cytidine deaminase APOBEC1, A3A-PBE is characterized by a wider deamination window (positions 1–17 in the protospacer) and independence from the sequence context (comparable activity in a TC and GC context) (Zong et al. 2018). The A3A-PBE was tested in transfected cv. Désirée protoplasts using multiple sgRNAs that targeted *StALS* or *StGBSS.* The human APOBEC3A was 11-fold more effective than the rat APOBEC1 in C-to-T editing. A high precision of A3A-PBE-mediated editing with a very low frequency of indels was demonstrated for both *StALS* and *StGBSS* in the transfected potato protoplasts and additionally confirmed in two independent heterozygous *StGBSS* mutant plants regenerated from protoplasts (Zong et al. 2018).

Several other studies have also demonstrated successful BE in potato. In the first approach, described in Veillet et al. (2019b, a), sgRNAs targeting either *StALS* or *StGBSS* were cloned into a binary vector carrying the CBE construct previously used for tomato editing (Shimatani et al. 2017). Using *StALS* as a target for BE allowed for the verification of the effectiveness of mutagenesis at the phenotype level and was useful for selecting non-transgenic BE events (Veillet et al. 2019b; see the following section). For *StGBSS* editing, the CBE vectors carrying two various sgRNAs targeting the gene regions encoding domains crucial for enzymatic activity were used for the stable *Agrobacterium*-mediated transformation of Désirée plants (Veillet et al. 2019a). High-resolution melting (HRM) analysis of the regenerated plants showed a remarkably high mutation efficiency of close to 90% (Veillet et al. 2019a). These results were confirmed by direct Sanger sequencing which also showed that Cs located in the CBE edition window were preferentially substituted with T and, albeit to a lesser extent, with G or A. However, in the analyzed sequences, indels were found frequently (77% for one of the sgRNAs), which strongly indicates the need for further optimization of the proposed CBE system for potato editing (Veillet et al. 2019a). Two Désirée mutants carrying the precise C17-to-G17 conversion (counting from the PAM), resulting in the L99V mutation within the KTGGL motif in StGBSS, may be used as research models for further studies of the functional and structural relationships in the StGBSS. This study proved the importance of the leucine residue in the KTGGL for StGBSS protein activity (Veillet et al. 2019a). The second approach (Veillet et al. 2020b) employed CBE constructs based on SpnCas9-NG (D10A), a novel nickase recognizing the non-canonical NGN PAM (Nishimasu et al. 2018). Two CBE construct variants, the first containing a single cytosine deaminase PmCDA1 and the second, a double CBE which consisted of human APOBEC3A (Zong et al. 2018), were used for stable transformation of the cv. Désirée. While the results were inconclusive, and indicate the need to optimize the presented method, there was an interesting finding: among the plants transformed with the double CBE, one harbored a C-to-T and another a C-to-G conversion at a distance of 13 and 11 nucleotides from PAM, respectively, which indicates that the CBE fused with human APOBEC3A may expand the editing window at NGN PAMs (Veillet et al. 2020b).

Recently, the application of PE tools in potato has been reported (Perroud et al. 2022). Although the proposed PE system showed exceptionally low editing rates, this study indicates that it is possible to introduce nucleotide transversion precisely into targeted potato genes. Perroud et al. (2022) assessed the effectivities of two different PE strategies (Anzalone et al. 2019) in the cv. Désirée and the moss *Physcomitrium patens*, a simple haploid model plant. The first, simplest PE strategy relied on the co-expression of genes encoding the fusion protein (prime editor), composed of SpnCas9 (H84A) and a Moloney murine leukemia virus reverse transcriptase (M-MLV RT), with a single pegRNA in the target cells. In the second strategy, an additional gene encoding standard sgRNA was co-expressed with the genes of the prime editor and the pegRNA (Perroud et al. 2022). The use of the additional sgRNA, which targeted the opposite strand of the edit site, aimed at enhancing the effectiveness of PE through the induction of an extra nick in the vicinity of the edited locus (Perroud et al. 2022). While both applied PE strategies showed high efficiency in editing *P. patens*, in the potato model, both were highly ineffective, and only one prime edited potato line was generated. However, three expected substitutions at the *StALS1* target locus were detected in the regenerated plant (Perroud et al. 2022).

#### Developing strategies for the generation of non-transgenic genome-edited potato

Due to the strong public resistance and complex approval procedures for GM crops, extensive research has been conducted with the goal of optimizing techniques for generating non-GM-edited plants. Since the most popular strategies to obtain non-transgenic edited plants, which rely on crossing and selection methods, are not useful for editing and releasing existing potato varieties, CRISPR/Cas technology offers new prospects for development in this field. As shown in various studies, the stable integration of the genes encoding CRISPR/Cas components is not necessary to achieve sufficiently high Cas nuclease activity to induce targeted mutations (e.g., Andersson et al. 2017; Chen et al. 2018; Veillet et al. 2019a; Bánfalvi et al. 2020; Perroud et al. 2022). Additionally, the lack of stable integration of the CRISPR/Cas genes reduces the probability of unwanted mutations caused by transgenes randomly inserted into the host genome. Thus, techniques for the transient activation of CRISPR/Cas components in the target cells have the potential to effectively generate non-GM edited potato plants (Gong et al. 2021). Interestingly, successful procedures for generating the non-GM potato lines with full allelic knockouts have been described for both *Agrobacterium*-mediated delivery of CRISPR/Cas components and protoplast transfection.

A short-term positive selection after *Agrobacterium*-mediated transformation is one of the methods proposed to induce transient CRISPR/Cas gene expression (Chen et al. 2018; Veillet et al. 2019a; Bánfalvi et al. 2020; Perroud et al. 2022). The approach is sufficient to successfully activate the Cas editing function, while significantly reducing the frequency of stable transformation events. Bánfalvi et al. (2020) showed that 3-day positive selection with kanamycin after *Agrobacterium*-mediated transformation with the CRISPR/Cas vector targeting *PDS* was sufficient to generate mutated potato shoots with a frequency of 2–10%. Analysis of 10 lines obtained after 3-day selection confirmed no integration of the CRISPR/Cas gene construct in the host genome (Bánfalvi et al. 2020). A more widely practiced approach is to extend the time of the positive selection treatment to 10–14 days (Chen et al. 2018; Veillet et al. 2019a; Bánfalvi et al. 2020; Perroud et al. 2022). To enhance the frequency of regenerated non-transgenic edited potato plants, the additional negative selection agent can be useful. For this purpose, the insertion of *CodA* gene, encoding an enzyme converting the non-toxic fluorocytosine (5-FC) to cytotoxic 5-fluorouracil, into the CRISPR/Cas plasmid was proposed. The addition of 5-FC to the regeneration medium allows the survival of only those events in which *CodA* has not been stably integrated into the genome (Bánfalvi et al. 2020). The negative selection approach can also be used when the SSN-mediated mutation of the endogenous gene results in the generation of plant lines resistant to a selection agent. For example, treatment with chlorsulfuron was applied to regenerate the potato lines with precisely edited Pro-189 residue in StALS (proline replacement with leucine or serine), using CBE and PE tools (Veillet et al. 2019b; Perroud et al. 2022)*.*

The techniques for protoplast transfection allow for the temporary activation of CRISPR/Cas elements in target cells. The transient expression system based on transfected protoplasts can be useful to rapidly and efficiently test the functionality of designed CRISPR/Cas constructs (Lukan et al. 2022) and is frequently used for the generation of transgene-free genome-edited plants (Fig. 1, left panel) (Zong et al. 2018; Fossi et al. 2019; Chauvin et al. 2021; Nicolia et al. 2021; Rather et al. 2022). In the case of potato, protocols for efficient protoplast isolation and transfection, followed by the regeneration of whole plants, are available (Nicolia et al. 2015, 2021; Andersson et al. 2017; Veillet et al. 2019a; González et al. 2020). Previous studies have demonstrated that the transient expression of CRISPR/Cas9 genes in protoplasts was sufficient to generate targeted mutations in the tetraploid cvs. Kuras, Désirée, and Furia (Andersson et al. 2017; Zong et al. 2018; Veillet et al. 2019a; Perroud et al. 2022). To generate non-transgenic genome-edited potato lines, protoplast transfection can be performed using either the appropriate genetic constructs carrying genes encoding CRISPR/Cas components or by the application of synthetically preassembled complexes of the purified Cas9 protein and sgRNA (RNPs) (Andersson et al. 2017, 2018; Veillet et al. 2019a; González et al. 2020, 2021; Chauvin et al. 2021). Comparison of genome editing using *Agrobacterium*-mediated transformation and both protoplast transfection-based methods to target *StPPO2* indicated a significantly higher mutation frequency in the events regenerated from protoplasts temporarily expressing CRISPR/Cas genes (31.9% editing efficiency) than in the events regenerated from the RNP-treated protoplasts or the agrotransformed explants (18.4 and 9.6% editing efficiency, respectively) (González et al. 2021). Moreover, only in the case of plants regenerated from the protoplasts transiently producing CRISPR/Cas components, the four allelic *StPPO2* mutations were observed. Mutation screening with high-resolution fragment analysis (HRFA) detected the full allelic mutations in 46% of the edited lines (González et al. 2021). In contrast, targeting *StGBSS* using the RNP method resulted in mutations in all four alleles in 2–3% of the regenerated shoots (Andersson et al. 2018).

Although protoplasts transfected with CRISPR/Cas components can be a promising model for functional gene studies, there are limitations to this system that should be considered. One limitation is that protoplast transfection with CRISPR/Cas plasmids may result in the insertion of DNA fragments (a few to several hundred bp) derived from degraded plasmids used for transfection. These types of foreign DNA fragments have been found both in the target sequences and at random sites of the host genome (Clasen et al. 2016; Andersson et al. 2018; Veillet et al. 2019a; González et al. 2020). However, this problem can be avoided if CRISPR/Cas RNPs are used for protoplast transfection (Andersson et al. 2018). Another important factor limiting the wide application of protoplast transfection in plant genome editing is the risk of inducing extensive somaclonal changes in the regenerated lines, as recently shown for potato (Fossi et al. 2019). Strategies to mitigate this risk might be based on the approach recently used for rice, which was successfully edited by CRISPR/Cas plasmids that had been transiently introduced into the zygote (Toda et al. 2019; Toda and Okamoto 2020). In planta agroinfiltration and strategies based on virus-induced genome engineering (VIGE) may be other valuable techniques in delivering CRISPR/Cas components (Nekrasov et al. 2013; Chincinska 2021; Uranga et al. 2021; Gentzel et al. 2022). So far, agroinfiltration has been successfully used to edit *Nicotiana benthamiana* and *S. tuberosum* cv. Russet Burbank and Shepody, using CRISPR/Cas and TALENs, respectively (Nekrasov et al. 2013; Ma et al. 2017). However, while effective procedures based on VIGE have been described for various plant species, the editing methods are not optimized for potato plants as of yet (Tuncel and Qi 2022).

### Potato as a food and industrial crop

Strategies for improving potato cultivars intended for consumption and industrial applications mainly focus on tuber yield and quality. Both traits highly depend on the genotype and the environmental conditions. To obtain the target genotype, breeding strategies that allow for the integration of new genome-editing technologies with available potato genetic resources are highly desired. The first step to achieving an efficient transfer of a beneficial genetic background into the cultivated potato is the CRISPR/Cas-mediated removal of self-incompatibility, which prevents obtaining homozygous inbred parents. Other successful potato improvement strategies using CRISPR/Cas editing include the enhancement of resistance to biotic stress, increasing the tolerance to CIS and EB, reducing the steroidal glycoalkaloid (SGA) content, and modifying the starch composition.

#### Generation of self-compatible diploid potato

Recently, a new approach in potato breeding has been proposed that would reinvent potato varieties as diploid inbred lines propagated by True Potato Seeds (TPS) (Jansky et al. 2016). There are several advantages to propagation by TPS, with perhaps the greatest being their near complete absence of pathogens (except the potato spindle tuber viroid, PSTVd). This makes TPS propagation particularly useful in developing countries since it allows for the reduction of costs generated by the production of pathogen-free clonally propagated tubers (Jansky et al. 2016). In addition, F1 inbred lines have been successfully used in the improvement of important agronomic traits in rice, maize (*Zea mays* L.), wheat, tomato, and soybean (*Glycine max* (L.) Merr.) but have never been used in potato due to several limitations. In the highly heterozygous tetraploid potato, achieving 99% homozygosity by self-pollination may take decades and is economically unprofitable. It is, therefore, much easier to employ diploid wild relatives to produce inbred lines. However, most of them are characterized by gametophytic self-incompatibility (Pushkarnath 1953; Pandey 1962).

Self-incompatibility is governed by a single multiallelic S-locus composed of *S-haplotype-specific F-box brothers* genes of *S-RNase* and *S-locus F-box* (*SLFs*) (Sijacic et al. 2004; Kubo et al. 2010). Both genes are expressed in the style and pollen. The S-RNase protein is responsible for self-pollen recognition and inhibition of its elongation through RNA degradation, whereas SLF redirects non-self S-RNase to the proteasome pathway and allows for pollen tube growth (Hua et al. 2008).

Restoration of self-compatibility in diploid potato is a valuable strategy in developing homozygous lines. Inhibition of the *S-RNase* function has been used to obtain self-compatible lines, stable in T0 and T1 generations (Ye et al. 2018; Enciso-Rodriguez et al. 2019). Due to the multiallelic *S-RNase* locus, sgRNA was designed to target the conserved domains of the S-RNase protein, encoded by the first (Ye et al. 2018) or first and second *S-RNase* exons (Enciso-Rodriguez et al. 2019). A total of five *S-RNase* alleles were targeted using the CRISPR/Cas9 method. The *Sp5*, *St5*, and *St6* alleles were identified in diploid potato lines generated from a cross of *S. tuberosum* group Phureja (according to current taxonomy, group Andigenum) and *S. tuberosum* group Tuberosum (according to current taxonomy, group Chilotanum) (Enciso-Rodriguez et al. 2019), while *Sp3* and *Sp4* in the *S. tuberosum* Gp. Phureja clone (Ye et al. 2018). Simultaneous knockout of the *Sp5*, *St5*, and *St6* alleles or *Sp3* and *Sp4* alleles resulted in mutation in at least one *S-RNase* allele, which was sufficient to obtain self-compatible diploid lines. In both studies, the formation of premature stop codons due to frameshift resulted in truncated S-RNase proteins and in the loss of their function in the recognition of self-compatible pollen, leading to self-compatibility (Ye et al. 2018; Enciso-Rodriguez et al. 2019).

This new strategy for the development of self-fertile diploid potato have paved the way for the production of inbred lines that contribute to improving progress in potato breeding. Moreover, self-fertile potato may provide a foundation for developing introgression lines, especially useful in the gene pool enrichment of the cultivated potato.

#### Enhancing biotic and abiotic stress resistance

*Phytophthora infestans* is one of the most devastating potato pathogens worldwide. Late blight symptoms include the formation of necrotic lesions, which may spread and cover the entire leaf surface and lead to plant death (Fry 2008). The predominant protection method against late blight is still the application of fungicides, although this exposes the environment to chemical pollution. A rational solution introduced decades ago was to obtain resistant varieties, which would minimize the use of fungicides. Resistance to *P. infestans* is determined by dominant *R* genes encoding proteins that can recognize avirulence (Avr) effectors and trigger plant responses (Fry 2016). However, the co-evolution of pathogen *Avr* genes and host plant *R* genes contributes to the durability of *R* gene-mediated resistance. Thus, the resistance of cultivars carrying only one *R*-gene is quickly overcome by new strains of *P. infestans*. Incorporation of the *R* gene from wild potato relatives and their pyramiding is currently the most reliable and environmentally friendly approach, albeit a time-consuming one (Stefańczyk et al. 2020), and indeed, *R* gene transfer has been successful (Halterman et al. 2015; Ghislain et al. 2019; Byarugaba et al. 2021). The resulting GM cultivars, resistant to a broad spectrum of *P. infestans* strains, have been intended mainly for cultivation in sub-Saharan Africa (Ghislain et al. 2019; Byarugaba et al. 2021).

CRISPR/Cas-based technologies have opened a new chapter in the field of the exploitation of plant resistance to *P. infestans*. This genome-editing tool has been used to increase potato resistance without manipulation within *R* genes. Two strategies have been applied to obtain plants with enhanced resistance: a knockin of the metabolite biosynthetic genes and a knockout of susceptibility genes (Hegde et al. 2021; Kieu et al. 2021).

The knockin approach was employed by Hegde et al. (2020, 2021) to enhance resistance to *P. infestans* in the cv. Russet Burbank. In their first study, they selected a pathogen-responsive gene, *StCCoAOMT*, based on an RNA-seq analysis of the gene expression profile of both mock and *P. infestans*-inoculated potato plants. The *StCCoAOMT* gene encodes caffeoyl-CoA methyltransferase, an enzyme that converts caffeoyl-CoA to feruloyl-CoA and produces hydroxycinnamic acids (Hegde et al. 2020). Interestingly, a specific allele of *StCCoAOMT* identified in Russet Burbank carries a point mutation that creates a premature termination codon; this results in a truncated protein lacking 96 amino acids from the N-terminus (Hegde et al. 2020, 2021). Using CRISPR/Cas9-mediated HDR, this nonsense mutation was replaced in all four *StCCoAOMT* alleles. This successfully restored resistance, which manifested as a significant reduction in plant severity and pathogen biomass (more than a 21-fold decrease) in stems. Additionally, an increased accumulation of feruloylated metabolites, involved in suberization and lignification of cell walls, was noted around the infection side (Hegde et al. 2021).

The second strategy to improve potato resistance to *P. infestans* employed an innovative approach based on the CRISPR/Cas9-mediated knockout of S-genes and the induction of recessive resistance (Kieu et al. 2021). Seven selected putative potato S-genes were analyzed in the cvs. Désirée and King Edward. Tetra-allelic deletion mutants were evaluated based on their resistance to *P. infestans* measured by lesion size, percentage of infected leaves, and seedling morphology. Three target S-genes, *StDND1*, *StCHL1*, and *StDMR6-1,* were shown to be involved in *P. infestans* susceptibility. Both *StCHL1* and *StDMR6-1* mutant plants were characterized by late blight resistance, demonstrating a reduced lesion size in comparison to the wild type (WT) and an unaffected morphology. These two lines with loss-of-function mutations of two S-genes may be used in breeding programs to introduce resistance to *P. infestans*, especially when combined with specific R-genes (Kieu et al. 2021).

The potyvirus PVY is the most destructive viral pathogen of potato. There are several PVY strains, classified according to a host response, which can range from barely noticeable symptoms on leaves, such as mosaic lesions and leaf drop (e.g., PVY^O^, PVY^C^), to severe symptoms such as tuber necrotic ringspot (e.g., PVY^NTN^) (Schubert et al. 2007). As PVY is transmitted to potato plants by various aphid species, the most common method of preventing the spread of the virus is the application of pesticides to reduce the vector population. The second and most effective method is the creation of resistant cultivars through conventional breeding or genetic engineering. Resistance to PVY is governed by R genes, e.g., *Ry*_*sto*_*, Ry-f*_*sto*_, and has been introduced into conventional cultivars mainly from *S. stoloniferum* (Flis et al. 2005; Szajko et al. 2008, 2014). However, as the PVY genome is prone to mutations, new strains can easily break down the resistance.

Recently, multiple PVY strain resistance has been successfully generated through the knockout of viral RNA transcripts. Four specific targets within conserved PVY genome regions were selected to construct vectors harboring the CRISPR/Cas13a cassettes containing sgRNAs and *LshCas13a* gene from *Leptotrichia shahii* driven by the *UBQ10* promoter (Zhan et al. 2019). Selected targets encode proteins responsible for virus replication and systemic infection (P3), virus movement (Cl), virus replication (Nlb), and virion assembly and systemic movement (CP). All potato lines with Cas13a/sgRNA targeting *P3*, *Cl*, *Nlb*, and *CP* in the PVY genome had the ability to cleave the RNA genome of PVY. Further, they showed no disease symptoms and a significant reduction of PVY^O^ and PVY^N^ accumulation in leaves, and the degree of PVY inhibition was positively correlated with the expression level of *LshCas13a/sgRNA* (Zhan et al. 2019).

In contrast to several reports confirming the usefulness of the CRISPR/Cas system in enhancing potato tolerance to biotic stresses, the application of this technology to generate abiotic stress-resistant varieties is still limited. A study by Zhou et al. (2017) on the molecular basis of the potato response to phosphate deficiency demonstrated that *StMYB44* suppresses the expression of the *StPHO1* gene and thus acts as a negative regulator of phosphate root-to-shoot transport. However, the potato lines with CRISPR/Cas9-mediated knockout of *StMYB44* did not show an increased phosphate content in roots and shoots compared to the WT (Zhou et al. 2017). CRISPR/Cas9 technology was also used for investigating the role of the transcription factor CYCLING DOF FACTOR 1 (StCDF1) and its natural antisense transcript (*StFLORE*) in drought stress responses (Ramírez Gonzales et al. 2021).

#### Reducing cold-induced sweetening

Long-term tuber storage requires a low temperature to sustain their quality and processing utility. However, low temperatures (approximately 4 °C) induce amylolytic enzymes, which break starch into the reducing sugars glucose and fructose, consequently decreasing potato chipping quality. During thermal processing, high RS levels together with asparagine (acrylamide precursor) contribute to the dark coloration of French fries and chips, which is not desirable for consumers (Kumar et al. 2004). The main enzymes responsible for the breakdown of sucrose derived from starch degradation are invertases, which occur in the form of isoenzymes localized in the cell wall, the vacuole (acid invertases), and the cytoplasm (alkaline invertases) (Draffehn et al. 2010). In potato, there are five genes encoding acid invertases, and one of these, encoding VInv, is expressed in tubers and is a target gene in CIS-related studies (Draffehn et al. 2010). Silencing the *VInv* gene in potato has previously been achieved mainly through the RNAi (Zhu et al. 2016) and TALEN (Clasen et al. 2016) technology, resulting in a highly reduced RS content.

Recently, CRISPR/Cas technology has been employed to reduce the RS content in potato tubers and the formation of acrylamide during frying by targeting *VInv* expression (Shumbe et al. 2020). VInv activity is regulated at the transcriptional level by cytosine methylation in the 1.0–1.7-kb promoter region and the post-translational level by invertase inhibitors (McKenzie et al. 2013; Shumbe et al. 2020). In the recently published preprint (Shumbe et al. 2020), the CRISPR-dCas9-DRM2 technology, which is based on the DNA-binding ability of dCas9 and DNA methyltransferase activity (Vojta et al. 2016; Papikian et al. 2019), was used for directed DNA methylation. De novo DNA-cytosine methylation within the *VInv* promoter significantly reduced its expression. The higher percentage of methylated cytosines corresponded to the lower RS accumulation in tubers (Shumbe et al. 2020).

#### Increasing tolerance to enzymatic browning

High tuber quality is one of the desired traits in the potato-processing industry and for consumers. The phenomenon known as EB contributes to the loss of nutritional quality and changes tuber taste and texture. It occurs when tubers are exposed to processing such as cutting, slicing, and peeling (Martinez and Whitaker 1995). Mechanical damage leads to a loss of the subcellular compartmentation and the release of amyloplast-localized PPOs and vacuole-localized phenolic compounds (Thygesen et al. 1995). In the presence of oxygen, PPOs catalyze the oxidation of monophenols and/or *o*-diphenols to *o*-quinones, which subsequently polymerize and form complexes with proteins, resulting in brown pigment accumulation (Thygesen et al. 1995; Boeckx et al. 2015). There are several chemical approaches to the prevention of EB in potato, most of which are based on PPO inhibition (Moon et al. 2020). The PPO is encoded by a multi-gene family, and in potato, five PPO genes have been identified so far, with several allele variations in each gene (Thygesen et al. 1995). Although EB is a quantitative trait, *PPOs* are common target genes to reduce EB in tubers since the PPO loci show a high association with the QTL, and the discoloration score depends on the allele combination (Werij et al. 2007). Since PPOs take part in many physiological processes and defense against pathogens and pests, knockout of all *PPO* genes would probably be lethal for plants. Therefore, precise gene targeting or allele silencing is required (Thygesen et al. 1995).

The *StPPO2* gene was successfully mutated in the cv. Désirée after CRISPR/Cas9 RNP delivery to protoplasts (González et al. 2020). The four-allele *StPPO2* edition produced two types of edited lines: with the frameshift mutations in the coding sequence and with the 111-bp non-frameshift mutation, which might have affected enzyme functionality after translation. Regardless of the mutation type, all lines were characterized by reduced *PPO* expression levels and lower susceptibility to EB in relation to the WT (González et al. 2020).

#### Decreasing the steroidal alkaloid content

Glycoalkaloids are secondary metabolites of solanaceous plants. More than 80 SGAs have been identified in potato species, among which α-solanine and α-chaconine are the most widespread ones in the cultivated potato (Ginzberg et al. 2009). The SGA accumulation in tissues is genotype- and environment-dependent. Due to their neurotoxic and anti-nutritional properties, as well as their contribution to the bitter taste of tubers, the level of SGAs in tubers intended for consumption should not exceed 200 mg kg^−1^ fresh weight (Valkonen et al. 2010). Since SGAs pose the first line of defense against pathogens and herbivores and take part in ecological interactions between plants and microorganisms, the complete elimination of SGAs would be detrimental to the plant itself. Nevertheless, substantial effort has been made to decrease the SGA content, focusing mainly on the appropriate selection of parental components during crossbreeding. A lower SGA content was also achieved by RNAi silencing of *sterol side chain reductase 2* (*StSSR2*) (Sawai et al. 2014) and *GLYCOALKALOID METABOLISM 1* (GAME1) (Cárdenas et al. 2016) genes, both involved in the SGA biosynthetic pathway.

One of the studies on the usefulness of the CRISPR/Cas system for manipulating SGA levels in potato aimed at knocking out the gene encoding steroid 16α-hydroxylase (*St16DOX*), which occurs in the genome in a single copy (Nakayasu et al. 2018). Hairy roots culture of the cv. Mayqeen was employed as a system for the screening of multiple candidate sgRNAs targeting different exons of *St16DOX*. Among the 25 established transgenic hairy root lines, two lines contained no detectable α-solanine and α-chaconine and accumulated 22,26-dihydroxycholesterol, a substrate of St16DOX, suggesting the complete disruption of *St16DOX*. However, the whole plants have not been regenerated (Nakayasu et al. 2018).

Although the CRISPR/Cas-mediated knockout of *St16DOX* allowed for the generation of the mutated hairy root lines with the desired phenotype, devoid of α-solanine and α-chaconine, not all attempts of SGA reduction in potato were similarly effective. Manipulation of the SGA level in the cv. Atlantic was mediated by CRISPR/Cas9-editing of *StSSR2* (Zheng et al. 2021). Although the SGA content in leaves was reduced in all six tested mutant lines, in four of these lines, the SGA level in the tuber flesh was higher than that in the WT. Further analysis of two lines with significantly lower SGA contents in the tuber flesh and peels revealed a reduced expression of *StSSR2* and the modified expression pattern of other genes involved in the SGA biosynthesis pathway, such as *squalene synthase*, *GAME*, and *solanidine glycosyltransferases*, which might have resulted in a decreased SGA accumulation (Zheng et al. 2021).

#### Modification of starch composition

Potato is an important source of starch both for food purposes and industrial applications. Potato starch usually consists of amylose (around 20–30%) and amylopectin (around 70–80%) (Grommers and van der Krogt 2009; Fajardo et al. 2013). Compared to amylose, which is a linear polymer of glucose residues linked with α-1,4 glycosidic bonds, amylopectin has a highly branched structure, containing short α-1,4-linked glucose chains connected by α-1,6 linkages (Nazarian-Firouzabadi and Visser 2017). The amylose/amylopectin ratio influences both dietary and industrial starch properties (Karlsson et al. 2007). Therefore, genetic engineering strategies mainly focus on either obtaining amylose-free (waxy) potatoes or increasing the amylose content, which can be achieved by targeting selected genes involved in starch biosynthesis (Hofvander et al. 2004; Andersson et al. 2017). CRISPR/Cas technology proved to be an efficient tool for both approaches.

Amylose-free starch has an improved freeze–thaw stability and is therefore an important ingredient in frozen food production. In addition, amylopectin, due to its binding properties, is used in paper and adhesive industries (Slattery et al. 2000). Potatoes producing amylopectin starch can be generated by knocking out or silencing the gene encoding GBSS, which has been successfully achieved by radiation-induced mutations, antisense technology, RNAi, TALENs, and, recently, using CRISPR/Cas (Andersson et al. 2017; Kusano et al. 2018). Since CRISPR/Cas-generated waxy potato lines have potential for future commercialization, most studies have focused on methods that can produce non-transgenic plants, namely through the transient expression of the CRISPR/Cas system in the protoplasts (Andersson et al. 2017; Veillet et al. 2019a) and delivering CRISPR/Cas as pre-assembled RNPs to the protoplasts (Andersson et al. 2018). The first approach resulted in unintended insertions of the vector DNA in 10% of the regenerated lines of the cv. Kuras (Andersson et al. 2017), whereas the use of RNPs containing synthetically produced sgRNA (cr-RNPs) generated exclusively transgene-free lines (Andersson et al. 2018). Among the lines with multiple mutated alleles, only the line with all four *StGBSS* alleles knocked out accumulated pure amylopectin starch (Andersson et al. 2017). Interestingly, Veillet et al. (2019a) demonstrated that CBE-generated substitutions in the locus encoding the catalytic domain KTGGL of the potato GBSS are sufficient to produce a loss-of-function allele. Amylose-free or amylose-reduced lines were also obtained using the CRISPR/Cas system for the stable transformation of Sayaka (Kusano et al. 2018) and Yukon Gold (Toinga-Villafuerte et al. 2022) cultivars, as well as sweet potato (Wang et al. 2019). However, due to public concerns over GM food, such transgenic lines may rather be considered as feedstock for industrial applications.

An elevated amylose content results in higher levels of resistant starch. Due to its slower digestion, it has a low glycemic index and can be fermented by gut bacteria, thereby producing health-beneficial short-chain fatty acids (Jobling 2004; Zhao et al. 2021). In the industry, high-amylose starch is used as a gelling agent and in the production of fried products (Slattery et al. 2000). Potato lines with increased amounts of amylose were generated via RNAi-mediated silencing of genes encoding starch-branching enzymes (SBEs) (Andersson et al. 2006) and thus studies using the CRISPR/Cas gene editing followed the same approach. For example, Tuncel et al. (2019) targeted *SBE1* and/or *SBE2* in the cv. Désirée using either *Agrobacterium*-mediated transformation or transient protoplast transfection to deliver the CRISPR/Cas9 components. The obtained potato lines exhibited a large variety of phenotypes, including an extreme phenotype that resulted from a strong reduction of SBE1 and SBE2 activity. It was characterized by lower levels of short amylopectin chains and a decrease in branching frequency compared to the WT. Mutations in *SBE1* alone did not affect the structure of accumulated starch, whereas lines with mutated *SBE2* contained a large number of tiny starch granules (Tuncel et al. 2019). In further studies, Zhao et al. (2021) targeted the same genes using the CRISPR/Cas RNP-method. The lines with mutations in all alleles of both *SBE1* and *SBE2* synthesized starch with no detectable branching and had reduced tuber yield, tuber size, and tuber dry matter content (Zhao et al. 2021). Both reports showed that the transient expression of the sgRNA/Cas9 constructs and delivery of RNP complexes to the protoplasts are promising tools for generating transgene-free plants with mutations in *SBE* genes (Tuncel et al. 2019; Zhao et al. 2021). However, the transient application of plasmids harboring the CRISPR/Cas cassette and sgRNA to the protoplasts can result in unwanted insertions of the plasmid fragments, as shown by other studies (Andersson et al. 2017; Veillet et al. 2019a).

## Future prospects

As potato is one of the world’s leading food source, most research is focused on improving its nutritional traits as well as increasing its yield and safety (Hameed et al. 2018). CRISPR/Cas technology, with emphasis on novel methods allowing for precise mutations such as PE and BE, seems to be highly promising in achieving these goals (Veillet et al. 2019a; Perroud et al. 2022). One of the priorities in the development of CRISPR/Cas editing procedures in potato is the establishment of methods for the generation of non-GM plants (Hameed et al. 2018; Nahirñak et al. 2022). This is crucial in the case of cultivars for food purposes, given the controversies as well as the subsequent lack of social acceptance regarding GM plants (Holme et al. 2019; Mir et al. 2022). The techniques enabling genome editing in planta appear to be particularly promising in achieving this goal (Chincinska 2021; Lei et al. 2021; Gentzel et al. 2022; Tuncel and Qi 2022). For example, a recently described editing of *N. benthamiana* used a dual-based vector system to co-express Cas12a nuclease gene and sgRNA (Uranga et al. 2021). The two compatible RNA virus vectors used in these studies were derived from tobacco etch virus (TEV) and PVX, and they offer a potential future application of this innovative VIGS system for editing the potato genome (Gentzel et al. 2022; Tuncel and Qi 2022). The possibility of the controlled introduction of mutations in the target plant genes using CRISPR/Cas techniques without the need to generate stably transformed lines can facilitate the commercialization of cultivars with favorable characteristics that have, so far, been generated only through stable genetic transformation.

The CRISPR/Cas application seems to be especially useful for traits determined by polygenes. Multiallelic, simultaneous knockout of various genes may improve the quality of tubers intended for the processing industry, e.g., increased tolerance to CIS, which was mainly achieved through the heterologous expression of *ADP-glucose pyrophosphorylase* (*AGPase*) (Stark et al. 1992), the silencing of *VInv* (Bhaskar et al. 2010), and the cis-transgenesis of starch phosphorylase L (Rommens et al. 2006). Studies on the simultaneous silencing of two genes encoding starch phosphorylase L and starch-associated R1, responsible for the starch breakdown, confirmed the high effectiveness of this approach in the reduction of the RS content and increasing the tolerance to CIS (Kamrani et al. 2016). Therefore, simultaneous knockout of the genes responsible for the starch breakdown and RS formation (acid invertases) creates new possibilities for generating CIS-tolerant potato.

Another direction in potato improvement using CRISPR/Cas deals with decreasing the SGA content in potato tubers. To date, not all CRISPR/Cas-mediated mutations of the selected candidate genes produced the desired effects at the phenotype level. Interestingly, some mutations even led to the opposite of the expected effect, as in the case of *StSSR2,* where some of the knockout lines had a higher SGA content than the WT (Zheng et al. 2021). For this reason, a precise selection of candidate genes is pivotal for successful editing. This is especially important in those metabolic pathways where the activity of one enzyme may be replaced by the activity of another enzyme (or isoenzyme). This also applies to SGA biosynthesis. As an alternative solution for reducing the SGA content, and a possible future application, CRISPR/Cas-mediated editing of *GAME* genes should be considered (Paudel et al. 2017). The *GAME* genes, which are involved in cholesterol conversion to SGA, neighbor each other on potato chromosomes and are organized in the form of metabolic gene clusters (Itkin et al. 2013). Some of these genes encode enzymes with similar activity, for example, *GAME2* (termed *SGT3* in potato), *GAME17*, and *GAME18* encode UDP-glycosyltransferases (Itkin et al. 2013). CRISPR/Cas technology for the multiallelic mutation could be applied to simultaneously knock down all three genes.

As potato is also used as a research model in plant molecular physiology, improving the effectiveness of CRISPR/Cas techniques for potato genome editing is expected to positively influence the development of functional genomics research. So far, RNA silencing technologies are the most successful approaches for functional gene studies in potato (Sharma et al. 2022). However, the application of target genome editing technologies, such as TALENs and CRISPR/Cas, which allow to generate a total (full allelic) gene knockout even in polyploid plants, may result in more unambiguous phenotypic effects compared to gene silencing at the transcription level (Guo et al. 2016; Wilson et al. 2019). Residual transcriptional activity of genes silenced using RNAi techniques can hinder or prevent a comprehensive understanding of their functions (Chincinska et al. 2008; Clasen et al. 2016; Guo et al. 2016). Hence, CRISPR/Cas techniques enabling precise changes at the genome level could be an excellent tool for elucidating the functions of potato genes, which, so far, have only been partially explained using other techniques of reverse genetics.

Improving the precision and efficiency of CRISPR/Cas procedures dedicated to potato genome editing can also have a significant impact on the growing interest in modifying potato tubers towards oil accumulation and in using potato as a potential bioreactor for recombinant protein production (Fig. 2, Biofactory). Increasing the triacylglycerol (TAG) content in potato tubers, which does not exceed 0.03% of dry weight, can improve both their nutritional value and their suitability as a raw material for industrial applications (Mitchell et al. 2017). Recent studies have demonstrated that it is possible to obtain transgenic potatoes accumulating TAGs up to 3.3% of tuber dry weight by overexpression of genes encoding a lipid-related transcription factor WRINKLED 1 (WRI1), TAG-synthesizing enzyme diacylglycerol acyltransferase 1 (DGAT1), and lipid droplet protein oleosin (OLEO1) (Hofvander et al. 2016; Liu et al. 2017; Mitchell et al. 2017; Xu et al. 2020). Additional RNAi-mediated downregulation of genes coding for AGPase, the first rate-limiting enzyme in starch biosynthesis, and the main TAG lipase sugar-dependent1 (SDP1) resulted in increasing TAG content in tubers up to 7% of dry weight (Xu et al. 2019). CRISPR/Cas technology can be used for further manipulation of the carbon flux not only to increase oil accumulation in potato but also to study the relationship between carbohydrate and lipid metabolism in tubers.

The potential of potato as a source of recombinant proteins has been widely discussed since the early 2000s, when potato tubers have been successfully used for the high-level production of a recombinant single-chain Fv and full-size immunoglobulin G, human serum albumin, human interleukin-2, or GP5 protein of the porcine reproductive and respiratory syndrome virus (Artsaenko et al. 1998; De Wilde et al. 2002; Park and Cheong 2002; Farran et al. 2002; Chen and Liu 2011). The great advantage of using potato as a biofactory is the possibility to store tubers containing recombinant proteins, retaining their active form for up to 6 months without a significant loss of content (Artsaenko et al. 1998; De Wilde et al. 2002). Such temporary storage of tubers accumulating recombinant proteins was postulated as a way to reduce the costs of transport and storage in the case of using potato for the production of therapeutics or vaccines, especially for developing countries, where access to proper cooling conditions could be limited (Artsaenko et al. 1998; De Wilde et al. 2002; Kim et al. 2008; Chen and Liu 2011). Among the obstacles hindering the implementation of such a recombinant protein production strategy are the high levels of glycoproteins belonging to the multi-gene family of patatins (Kim et al. 2008). High patatin content can not only hinder the heterologous overproduction of recombinant proteins in tubers but can also make it difficult to purify recombinant products (Kim et al. 2008). Using CRISPR/Cas technology to multi-gene patatin knockout in potato tubers could be a way around this problem. The effectiveness of such an approach has been demonstrated by previously conducted studies on RNAi potato lines, which showed reduced contents of patatin at the mRNA and protein level by about 99%. Despite such a significant reduction in patatin content, the RNAi plants showed no phenotypic differences related to their overall condition and tuber production compared to WT lines (Kim et al. 2008). Based on these results, it can be assumed that the full allelic patatin gene knockout at the genome level using CRISPR/Cas can be neutral for the general phenotype in potato plants. This would be significant for the intended use of the patatin-deficient tubers as future bioreactors for recombinant protein production.

As for other plant expression systems, the important step in adapting potato tubers to human protein production is the humanization of post-translational modifications, particularly glycosylation (Schneider et al. 2015). In previous studies, *N. benthamiana* RNAi lines with the targeted down-regulation of genes encoding the endogenous β1,2-xylosyltransferase and α1,3-fucosyltransferase could produce monoclonal antibodies containing almost homogeneous N-glycan species without detectable xylose and α1,3-fucose residues, which exhibited functional properties similar to those produced in mammalian and human expression systems (Strasser et al. 2008; Göritzer et al. 2017). The application of CRISPR/Cas methods to generate glycoengineered tubers would significantly increase the potential of using potato in molecular pharming as a source of humanized glycoproteins.

## *Author contribution statement*

IC, MM, and DSK: conceptualized the idea, prepared the background information, performed the literature survey, and critically evaluated the manuscript. All authors prepared the manuscript and read the final version, provided necessary suggestions, and approved it for publication.

## Data Availability

Not applicable.
